# The Design and Development of a Ship Trajectory Data Management and Analysis System Based on AIS

**DOI:** 10.3390/s22010310

**Published:** 2021-12-31

**Authors:** Chengxu Feng, Bing Fu, Yasong Luo, Houpu Li

**Affiliations:** 1 College of Weapon Engineering, Naval University of Engineering, Wuhan 430033, China; kerryfengcx@126.com (C.F.); 13476039901@139.com (B.F.); 2 College of Electrical Engineering, Naval University of Engineering, Wuhan 430033, China; Lihoupu1985@126.com

**Keywords:** AIS, ship trajectory, data analysis, system design, trajectory classification

## Abstract

To address the data storage, management, analysis, and mining of ship targets, the object-oriented method was employed to design the overall structure and functional modules of a ship trajectory data management and analysis system (STDMAS). This paper elaborates the detailed design and technical information of the system’s logical structure, module composition, physical deployment, and main functional modules such as database management, trajectory analysis, trajectory mining, and situation analysis. A ship identification method based on the motion features was put forward. With the method, ship trajectory was first partitioned into sub-trajectories in various behavioral patterns, and effective motion features were then extracted. Machine learning algorithms were utilized for training and testing to identify many types of ships. STDMAS implements such functions as database management, trajectory analysis, historical situation review, and ship identification and outlier detection based on trajectory classification. STDMAS can satisfy the practical needs for the data management, analysis, and mining of maritime targets because it is easy to apply, maintain, and expand.

## 1. Introduction

China faces an increasing demand for marine resources and ocean space along with economic and social development, bringing a variety of practical challenges to its maritime regulation and security. In the maritime regulation field, it has become more and more difficult to supervise and regulate the ships engaging in illegal fishing and maritime smuggling because these ships evade detection [1]. In the military and national security field, China’s national defense and military security is severely threatened by military vessels in disguise, including survey ships and electronic reconnaissance ships that some countries sent to deliberately perform illegal activities such as seabed and hydrological surveying and mapping and military reconnaissance in China’s offshore waters [2]. In the nontraditional maritime security field, China faces rampant criminal activities on its shipping routes across the South China Sea and the Strait of Malacca, etc., and the passing ships are also exposed to the serious threat posed by pirates, armed hijacking at sea, and maritime terrorism. Therefore, implementing an accurate classification of unknown ships, the effective identification of suspected ships, and the timely detection of ships doing abnormal activity is of great significance to achieving effective maritime regulation and defending maritime security [3].

Apart from further strengthening the maritime military forces and increasing patrols and law enforcement efforts, “soft methods” must also be employed to cope with these challenges, e.g., improving the automatic identification system (AIS) receiving stations and radar surveillance stations [3]. The data analysis in AIS can be conducted to dig out some valuable information from the spatial and temporal ship trajectory data that are constantly accumulated. Computers are able to automatically learn the regular movement and behavioral patterns of ships [2]. In this way, the analysis can assist maritime supervisors and military commanders in rapidly locating any suspected and abnormal target based on the massive quantities of information on ship trajectory. This analysis is then combined with other detection means to comprehensively judge the actual identity of suspected and abnormal maritime targets and their purposes.

In recent years, attention has been focused on the classification and identification of moving targets using data mining technology and AIS trajectory data. Reference [1] studied temporal and spatial data mining algorithms, and illustrated the design and implementation of a moving object activity pattern recognition system. The system utilized methods as data cleaning, data interpolation, and compression to preliminarily process the original trajectory data. Subsequently, a trajectory clustering algorithm was designed to realize the identification and classification of similar motion modes. Nevertheless, it overlooked the influence of time on the behavioral characteristics of ships, so that it was not applicable to the classification and identification of long-duration ships. Reference [2] put forward a ship behavioral pattern recognition method based on machine learning. In this method, AIS trajectory data were segmented to generate sub-trajectories for dimension reduction and visualization of data. Subsequently, sub-trajectories were clustered using the spectral clustering algorithm to recognize the behavioral pattern of ships. In reference [3], a new port ship classification method based on behavioral clustering was proposed to first conduct behavioral clustering of ships in a port and then extract the clustering characteristics. The ships were then classified into different behavioral clusters based on the ship features. The proposed method helped resolve the classification and identification of ships in the port, but was not applicable for ships sailing at sea. Reference [4] presented a ship classification model based on a graph neural network (GNN). The trajectory features extracted with the model contained such temporal and spatial features as location, distance, and speed. Additionally, a topologic correlation network was constructed to effectively extract the spatial features for ship classification and identification. Nevertheless, it did not consider the intrinsic differences between different types of moving objects.

This paper takes the AIS as a fundamental platform to design and develop a ship trajectory data management and analysis system (STDMAS) with various functions following the trajectory data management and analysis method. A ship identification method based on the motion features was put forward. With the method, ship trajectory was first partitioned into sub-trajectories in various behavioral patterns, and effective motion features were then extracted. Machine learning algorithms were utilized for training and testing to identify many types of ships. By addressing the practical needs of ship management and regulation, STDMAS can help realize the effective management of ship trajectory data, so as to effectively support and safeguard China’s maritime management and maritime security.

## 2. Demand and Functional Analysis

The main tasks of STDMAS include managing the storage of ship trajectory data from various sources to provide protection service for the historical data on spatial and temporal ship trajectory; assessing and analyzing the quality of trajectory data from various sources to provide the data support for evaluating the equipment performance based on the collected ship target trajectory data; and analyzing and mining the information on the hot spots and situation regularity in the waters around China to provide the technical support for the analysis and decision making in maritime supervision and management [5,6]. It can also analyze the navigational characteristics, pattern, and regularity of ships to overcome the impossible identification of many targets at sea and provide the basis for maritime administration and military commanders in the selection and tracking of key targets among many unknown ships [7].

**Figure 1:**
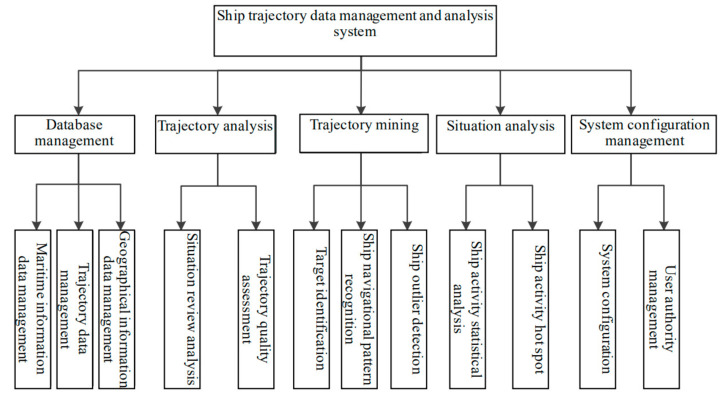
Main functional modules of STDMAS.

STDMAS has five modules: database management module, trajectory analysis module, trajectory mining module, situation analysis module, and configuration management module. Among them, the database management module can be further divided into three sub-modules including original maritime information data management, trajectory data management, and geographical information data management. The trajectory analysis module is further divided into two sub-modules: situation review analysis and trajectory quality assessment [8]. The trajectory mining module is further divided into three sub-modules, including target identification, ship navigational pattern detection, and ship outlier detection. The situation analysis module is further divided into two sub-modules, including ship activity statistical analysis and ship activity hot spots. The system configuration management module is further segmented into two sub-modules, i.e., system configuration and user authority management. The main functional modules of STDMAS are presented in Figure 1.

## 3. System Design

### 3.1. Overall Architecture

**Figure 2:**
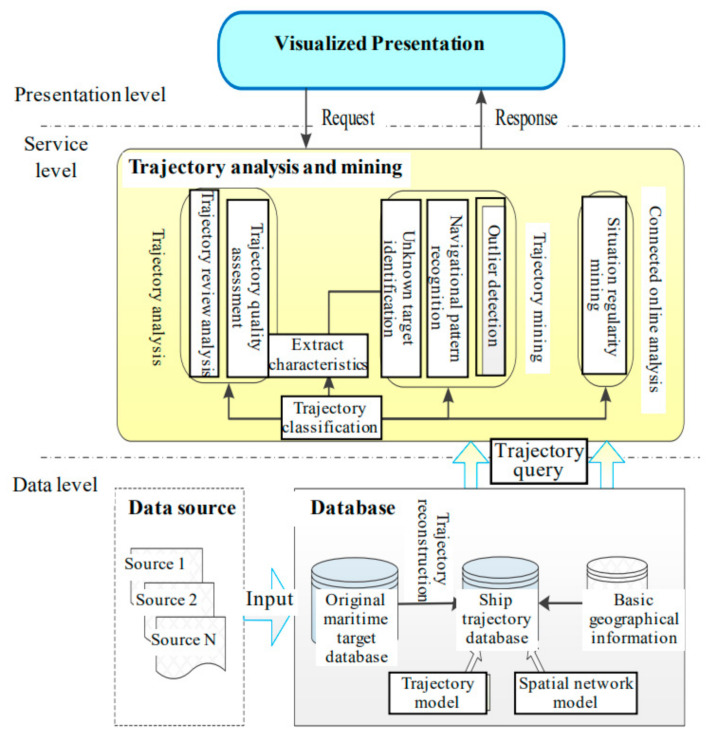
Logical structure diagram of STDMAS.

The logical structure of STDMAS is designed as shown in Figure 2. In general, the structure is divided into three levels, i.e., the data level, service level, and presentation level. The data level is mainly responsible for the storage, management, and maintenance of massive amounts of ship trajectory data, and it transmits the data to the service level. At the service level, the maritime data are sorted out and processed to remove invalid redundant data. Subsequently, a variety of algorithms are employed to obtain the regularity, pattern, and knowledge of ship navigation, which are directly provided to the presentation level or stored in the dataset. The presentation level allows for human-computer interaction so that a user request for computation service is sent to the service level to obtain the abstract data. After that, the abstract data are presented to the user in graphical form [9,10].

Data level: As the fundamental database of STDMAS, this level is mainly responsible for cleaning, filtering, and converting the massive multi-source heterogeneous data of maritime targets. Subsequently, it puts the maritime data into storage in a unified format and provides the corresponding interface for data management, maintenance, and query. For this level, attention is mainly paid to the marine geospatial representation model, trajectory data representation model, massive trajectory fast storage method, and fast temporal and spatial query method, etc.

Service level: As the business center of STDMAS, this level is responsible for the central computation service of trajectory data. It involves three modules, i.e., trajectory analysis, trajectory mining, and online analysis processing. It implements such functions as ship historical trajectory review and replay, data quality assessment of various information sources, ship identification, ship navigational regularity mining, ship outlier detection, and situation regularity mining in the waters around China [11].

Presentation level: This level is responsible for presenting the human-computer interaction operations and the results of data analysis. After borrowing the data presentation methods from other similar platforms and soliciting opinions from maritime information supervision users, the GIS display technique and graphic user interface technique are comprehensively applied to present the results of trajectory data computation and analysis to users in the form of maps, diagrams, and data reports.

### 3.2. Technical Route

For the design and development of STDMAS, the technical route is determined according to the requirements for system construction and functions. It also ensures that the system is easy to expand and maintain.

(1) The object-oriented method is employed to design the system. The unified modeling language UML is taken as the analysis tool to enhance the reuse rate of code.
(2) The system adopts the C/S architecture based on the Visual Studio 2013.NET development environment. It is developed with the C# programming language that is absolutely object-oriented.
(3) The GIS spatial display module is developed using SharpMap. As a completely open source and lightweight GIS rendering tool, SharpMap offers a variety of underlying function interfaces.
(4) The database is the open source object-relational database PostgreSQL with the PostGIS spatial module plug-in. Additionally, Npgsql dynamic link library is employed to connect the system with the database [12,13].

### 3.3. Physical Deployment

**Figure 3:**
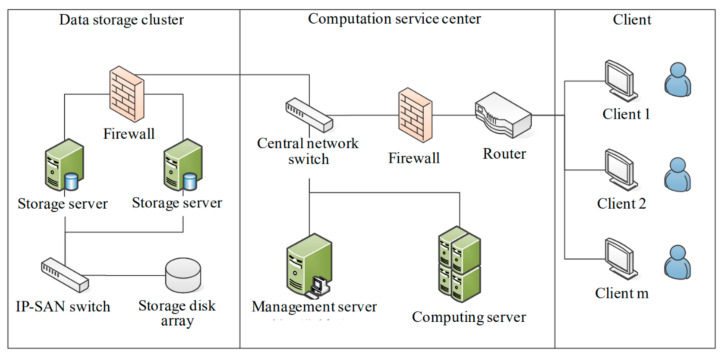
Physical deployment diagram of STDMAS.

The physical deployment of STDMAS, as shown in Figure 3, involves a data storage cluster, a computation service center, clients, and a network.

(1) The data storage cluster is the foundation of the entire system. The data from different information sources have different security levels. Therefore, physical separation is ensured in the storage of the maritime data from different sources to guarantee the absolute security and control of the data. For the efficient, stable, and expandable management of data storage, IP-SAN two-layer data storage architecture is employed, and the data storage disk array supports convenient future expansion. The storage capacity is 100 T.
(2) The computation service center is mainly composed of a switch, a router, a computing server, and a management server. The management server is responsible for system configuration and user authority management. The computing server is connected by high speed Ethernet to the data storage cluster. It allows querying and sorting of the maritime data and other operations through the storage server in the data storage cluster. In this way, it can fulfill such functions as maritime and air situation data loading management, storage management, data query, system review analysis, trajectory mining, and situation analysis. Through the client, a user can send a data analysis request to the computation service center. The computing server can then extract the data from the data storage cluster for computation, and transmit the computation results to the client [14].
(3) A client is a computer for any maritime data management user or common user in the local area network. Maritime data users can send a service request through a client to the computation service center, and obtain the corresponding trajectory data, graphic reports, or other views.
(4) The network in the system is divided into three parts, i.e., the part in the data storage cluster, the part in the computation service center, and the part for interaction between the computation service center and the client user. After comprehensively considering the needs for functions and the construction cost, optical fiber connection was provided in the data storage cluster (between the storage server and the storage disk array), whereas the Gigabit Ethernet was used in the computation service center [15]. A 100 m local area network is employed to connect the computation service center with the clients.

## 4. Design of Functional Modules

### 4.1. Database Management Module

**Figure 4:**
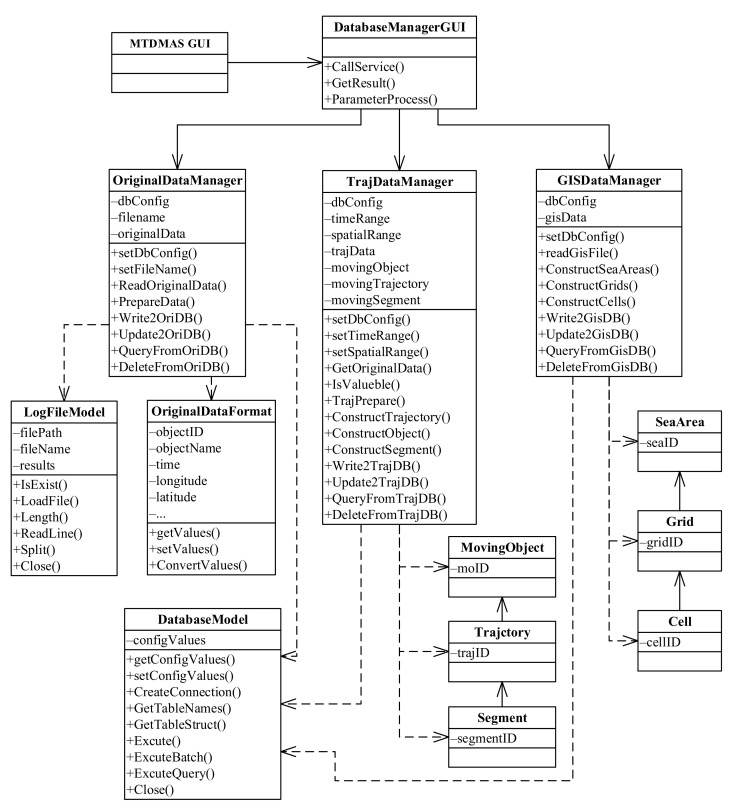
Class diagram of the database management module.

The database management module provides the basic data for the upper-layer services such as trajectory data analysis and mining [16]. The class diagram for the UML design of the module is presented in Figure 4. In the diagram, the GUI class of STDMAS represents the overall graphical user interface of the system. It can be used to call the graphical user interface of the database management module, i.e., the DatabaseManagerGUI class. The DatabaseManagerGUI class can call the functional sub-modules of the database management module, i.e., original maritime data management (OriginalDataManager class), trajectory data management (TrajDataManager class), and geographical information data management (GISDataManager class) [17].

The OriginalDataManger class implements the storage, query, deletion, and other management services of original maritime information data. The class mainly contains the object dbConfig indicating the database connection configuration, the object fileName indicating the file name of original maritime information data files, and the object originalData indicating the storage of original data. Among the methods used in the class, ReadOriginalData() is responsible for reading the original maritime data of different formats from various sources and depends on the LogFileModel class representing the reading of files; PrepareData() is used for the cleaning, format conversion, and other operations of the original maritime data after being read and relies on the OriginalDataFormat class for the unified format; Write2OriDB() implements the storage of the original maritime data and depends on the DatabaseModel class for realizing the basic database services. Moreover, the OriginalDataManger class can also, upon the user’s request, call the methods Update2OriDB(), QueryFromOriDB(), and DeleteFromOriDB() for the update, query, and deletion in the original maritime database.

The TrajDataManager class implements the storage, query, deletion, and other management services of trajectory data. The class mainly consists of the object dbConfig indicating the database connection configuration, the objects timeRange and spatialRange indicating the time and spatial range, respectively, selected by a user, the object trajData saving the constructed trajectory data, and other objects such as movingObject, movingTrajectory, and movingSegment. Among them, the objects movingObject, movingTrajectory, and movingSegment have their type of data depending on the MovingObject, Trajectory and Segment classes for the trajectory data model. Among the methods for the TrajDataManager class, GetOriginalData() is used to extract all the original data in the time and spatial ranges given by the user from the original maritime database; TrajPrepare() allows reconstructing the trajectory with all the trajectory data of a target, and IsValueble() is then employed to judge the validity of the trajectory, remove the invalid points, and smooth the trajectory; ConstructObject(), ConstructTrajectory(), and ConstructSegment() are used to construct the trajectory data model designed in this paper; Write2TrajDB(), Update2TrajDB(), QueryFromTrajDB(), and DeleteFromTrajDB() can store, update, query, and delete the data in the trajectory database upon a user’s request. Like those in the OriginalDataManger class, these methods also rely on the DatabaseModel class [18].

The GISDataManager class implements the storage, query, deletion, and other management services of geographical information data. The class is mainly composed of the object dbConfig indicating the database connection configuration, and the object gisData indicating the storage of geographical information data. In the class, readGisFile() is used to read the geographical information data, and the methods ConstructSeaAreas(), ConstructGrids(), and ConstructCells() are responsible for converting the geographical information data into the spatial grid model. The three methods rely on the SeaArea class, the Grid class, and the Cell class, respectively. The methods Write2GisDB(), Update2GisDB(), QueryFromGisDB(), and DeleteFromGisDB() can implement the storage, update, query, and deletion in the geographical information database upon a user’s request. Similarly, these methods are also dependent on the DatabaseModel model [19].

### 4.2. Trajectory Analysis Module

**Figure 5:**
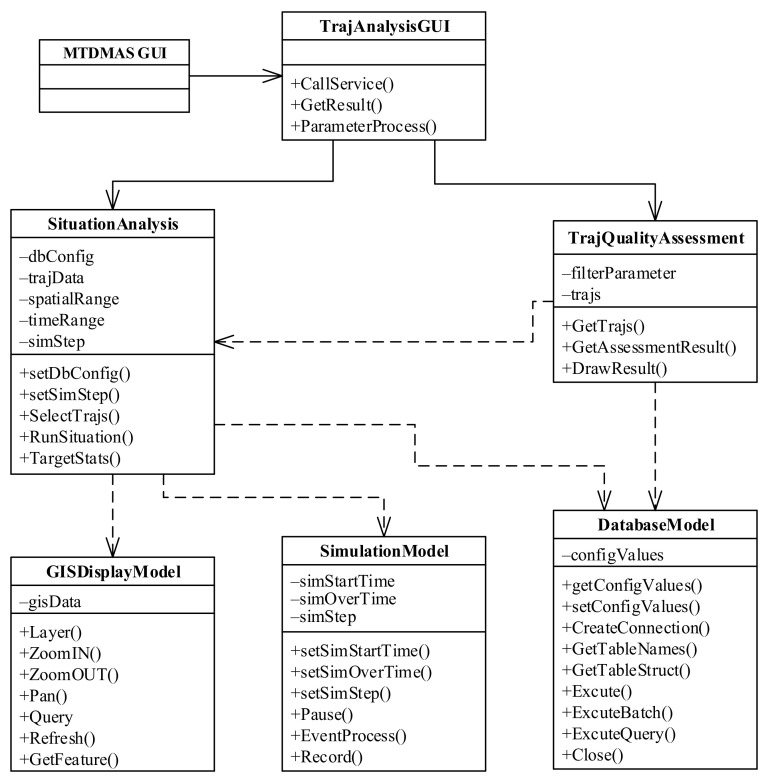
Class diagram of the trajectory analysis module.

The class diagram of the trajectory analysis module is shown in Figure 5. In the diagram, the TrajAnalysisGUI class represents the graphical user interface of the trajectory analysis module. It can also be called by virtue of the STDMAS GUI class. The graphical user interface (TrajAnalysisGUI) of the trajectory analysis module can be also used to call two functional sub-modules, i.e., situation review analysis (the SituationAnalysis class) and trajectory quality assessment (the TrajQualityAssessment class).

The SituationAnalysis class mainly implements such functions as querying all the maritime data in the time and spatial ranges given by a user, performing the review, replay, and analysis of maritime situation, creating the sequence of events for the sea and air situation in the corresponding process, and reproducing the maritime situation evolution process of missions and events in the corresponding time and spatial ranges. The SituationAnalysis class contains the object dbConfig indicating the database configuration, the objects spatialRange and timeRange indicating the time and spatial ranges selected by a user, the object simStep determining the speed of replay, and the object trajData for the storage and query of trajectory data. Among the main methods for the class, SelectTrajs() is used to check the trajectory in the trajectory database based on the time and spatial ranges given by a user, and depends on the DatabaseModel class; RunSituation() calculates the location of each target at each time of simulation by calling the simulation module (the SimulationModel class) and replays the maritime situation using the simulation step simStep through the geographical information display module (the GISDisplayModel class); TargetStats() is employed for the statistics of the information on various targets, e.g., quantity, and displays the information in the form of a report [20,21,22].

The TrajQualityAssessment class is designed to analyze and assess the quality of the selected trajectories. In the class, the object filterParameter indicates the conditions for selecting trajectories, and the object trajs stores all the selected trajectories. The method GetTrajs() can extract the trajectory data from the database or from the trajectories in the replay and analysis module. It depends on the DatabaseModel class and the SituationAnalysis class. The method GetAssessment() is used to analyze and assess the quality of the selected trajectories. The method DrawResult() displays the results of analysis and assessment to the user in graphical form. The indicators of the trajectory quality assessment include target identification rate, average identification response time, trajectory outlier rate, continuous tracking rate, and fault tracking rate [23].

### 4.3. Trajectory Mining Module

**Figure 6:**
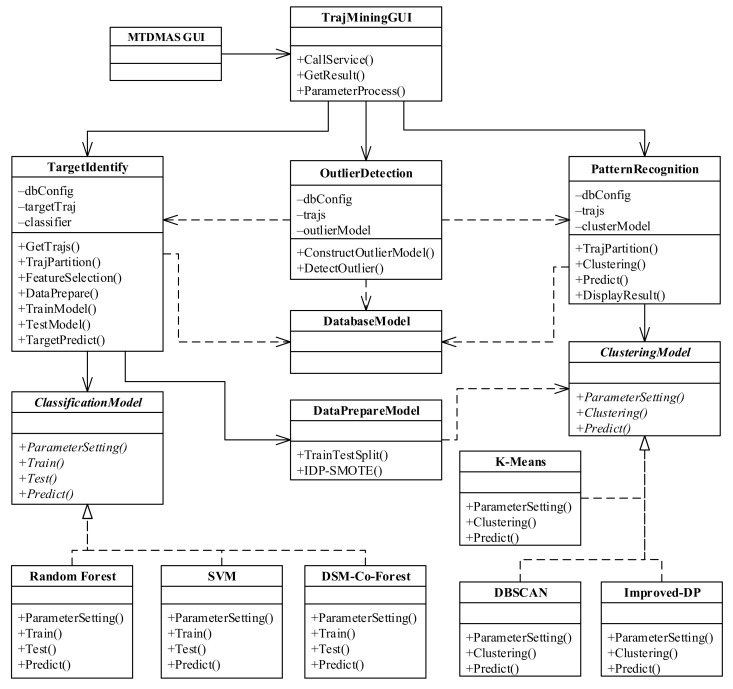
Class diagram of the trajectory mining module.

The UML class diagram of the trajectory mining module is shown in Figure 6. In the diagram, the TrajMiningGUI class represents the graphical user interface of the trajectory mining module, and can be called by the STDMAS GUI class. The TargetIdentify class, the OutlierDetection class, and the PatternRecognition class are used to implement three functions of the trajectory mining, respectively, that is, identifying the unknown ships, detecting the ship outliers, and recognizing the navigational pattern of ships. The OutlierDetection class mainly involves the methods ConstructOutlierModel() and DetectOutlier(), which are used to construct and detect the outlier model, respectively. The ship outlier detection model in the STDMAS depends on the identification of unknown ships (the TargetIdentify class) and the recognition of ship navigational patterns (the PatternRecognition class) [24].

The TargetIdentify class can construct the ship trajectory classification model based on the parameters set by a user and then use the model to determine the category of any unknown ship. The class consists of the database configuration object dbConfig, the trajectory data set targetTraj, and the classifier, etc. In the TargetIdentify class, the method GetTrajs() is employed to extract the trajectory data needed from the trajectory database according to the user’s request, and depends on the DatabaseModel class [25]. The method TrajPartition() can partition each ship trajectory using the proposed trajectory partitioning method based on the movement mode in this paper. Subsequently, the method FeatureSelection() is utilized to extract the characteristics of each trajectory in the way proposed in this paper. The method DataPrepare() aims to classify the characteristic data sets into the training set and test set and utilizes the algorithms including IDP-SMOTE to balance these data sets. The method depends on the DataPrepareModel class and indirectly on the clustering model, i.e., the ClusteringModel class [26]. TrainModel(), TestModel(), and TargetPredict() represent the training, test, and prediction of the classification model, respectively, and depend on the ClassificationModel class for the abstract classification model. It implements the RandomForest class for the random forest model, the SVM class for the support vector model, and the DSM-Co-Forest class for the semi-supervised learning model. Meanwhile, it can be further expanded based on the specific classification model.

The PatternRecognition class is used to construct the clustering model based on the trajectory selected by a user and display the trajectory clustering results in graphical form. The class consists of the objects dbConfig, trajs, and clusterModel [27]. The methods mainly include TrajPartition() for trajectory partitioning, Clustering() for trajectory clustering modeling, Predict() for cluster prediction, and DisplayResult() for displaying the trajectory clustering result. In the class, the construction of the cluster model depends on the abstract class, i.e., the ClusteringModel class, which implements the cluster models including the K-Means model, the DBSCAN, and the Improved-DP model.

### 4.4. Situation Analysis Module

**Figure 7:**
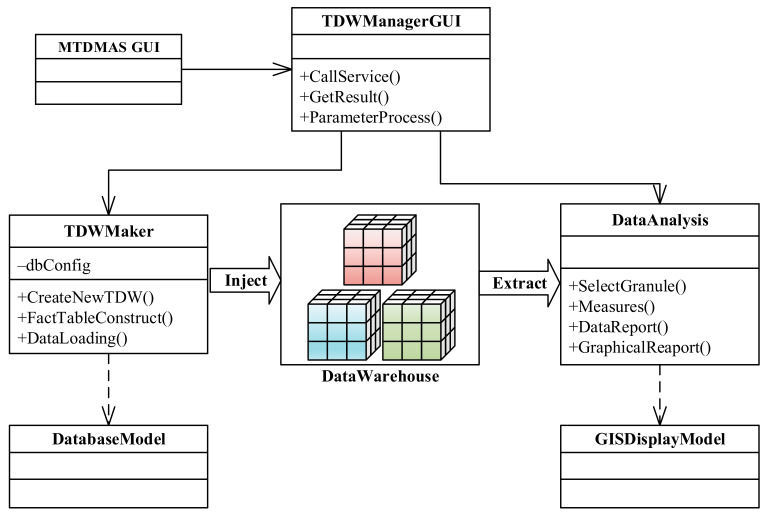
Class diagram of the situation analysis module.

The UML class diagram of the situation analysis module is shown in Figure 7. In the diagram, the TDWManagerGUI class represents the graphical user interface of the situation analysis module. It is also called through the STDMAS GUI class.

The TDWMaker class is mainly used to construct the trajectory data warehouse. Among its methods, CreateNewTDW() can create the new data mart for a new subject; FactTableConstruct() can define the fact table and dimension table based on the configuration given by a user; DataLoading() can extract the processed data from the trajectory database in the extraction-transformation-loading (ETL) method and then input the data into the data warehouse, but it depends on the DatabaseModel class for implementation [28].

The DataAnalysis class is mainly intended to extract information from the trajectory data warehouse upon a user’s request, and it generates the reports. Among its methods, SelectGranule() is used to select the granule of time, space, and other dimensions for data analysis; Measures() can further calculate and obtain the measured value of a subject (e.g., speed, distance, pass time, etc.) at each granule; DataReport() and GraphicalReport() can present the results of data analysis in the form of a data report or graphical report. However, the DataAnalysis class must rely on the GISDisplayModel class in the display of data analysis results, so as to more vividly present the changes of historical maritime situations.

## 5. Methods for Ship Trajectory Data Mining

Presently, trajectory mining technology offers four features in terms of mission and based on temporal and spatial characteristics, that is, trajectory pattern mining, trajectory clustering analysis, trajectory classification, and trajectory outlier detection [2]. Among them, the trajectory pattern mining feature discovers the valuable motion feature patterns for a single moving object or a group of moving objects, e.g., frequent pattern, periodic pattern, and adjoint pattern, so as to help people understand the motion regularity of the moving object and reasonably predict its future trend of motion. Trajectory clustering intends to devise a measure of similarity between trajectories and cluster the highly similar trajectories, in order to find out the representative path or common behavioral tendency of moving objects. Trajectory classification aims to predict the motion state of moving objects or their means of transport by extracting the features of trajectory or trajectory sections and then classifying them into different categories. Trajectory outlier detection can identify any suspected moving object or behavior based on the motion regularity or behavioral pattern of historical trajectories.

### 5.1. Ship Trajectory Partitioning

In the studies of trajectory motion mode, many scholars divide trajectory only into Stop Mode and Move Mode. However, ships at sea may take any turn for a specified reason or purpose unlike the moving objects on the ground, e.g., vehicles and pedestrians, which are restricted by road grids. In other words, turn is also an important motion mode of ship trajectory. On this basis, this paper partitions ship trajectory into three basic motion modes, that is, Stop Mode, Turn Mode, and Line Mode [7,8].

**Figure 8:**
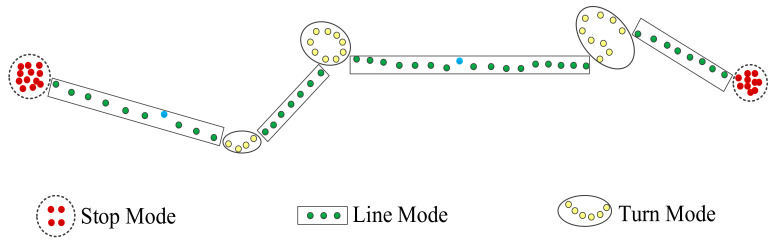
Three basic motion modes of a ship trajectory.

(1) Stop Mode: A ship trajectory contains a segment of continuous sub-trajectories in which the speed at all points is lower than the speed threshold. The distance between any two trajectory points must be lower than the distance threshold, and the duration is greater than the time threshold. At this time, the motion mode of the sub-trajectory is Stop Mode.
(2) Turn Mode: The turn threshold is set to $\Delta_{\theta }$. If the sum of trajectory direction changes within a range of time $\Delta_{t1}$ before and after a trajectory point p is greater than $\Delta_{\theta }$, the trajectory point p is a potential turn point. If the potential turn point is a segment of a continuous sub-trajectory, and the total time for such sub-trajectory exceeds the threshold $\Delta_{t2}$, the motion mode of the sub-trajectory is Stop Mode.
(3) Line Mode: In a ship trajectory, the remaining sub-trajectory is in the Line Mode after taking out those in the Stop Mode and the Turn Mode. The three motion modes of a ship trajectory are illustrated in Figure 8.

For further data mining and knowledge discovery, a ship trajectory can be partitioned in terms of these three basic motion modes in the following procedure:

Step 1: Partition a ship trajectory into several continuous sub-trajectory segments;

Step 2: Go through all the sub-trajectory segments. When the speed at most of the points in a sub-trajectory segment is less than the speed threshold $\delta_{v}$, it is judged that the sub-trajectory is in the Stop Mode. Otherwise, it is in the Move Mode;

Step 3: Go through all the sub-trajectory segments in the Move Mode. When the sum of direction changes at the points of a sub-trajectory within the time threshold exceeds the turn threshold, it is judged that the sub-trajectory is in the Turn Mode. Otherwise, it is in the Line Mode;

Step 4: Use the outlier detection algorithm to rule out the outliers in the trajectory;

Step 5: Add the segmented ship sub-trajectories into the corresponding sets of Stop Mode, Turn Mode, and Line Mode.

### 5.2. Ship Trajectory Feature Extraction

For higher efficiency of the trajectory data mining algorithm, the trajectory features must be selected in terms of their contribution to ship identification. Based on the above ship trajectory partitioning algorithm, trajectory features are classified into four categories, that is, global features, stop features, line features, and turn features.

(1) Global features refer to the extracted general features of the entire trajectory and its sub-trajectories and reflect the features of the trajectory holistically. There were nine global features extracted in this paper, including total sailing time, total sailing distance, total sailing sinuosity, number of sub-trajectories in the Stop Mode, Line Mode, and Turn Mode, and their respective proportion of total trajectory time. Among these global features, trajectory sinuosity represents the ratio of the sailing distance between two trajectory points and the straight distance between them and is employed to indicate the curvature of the path. Total trajectory sinuosity is the ratio of the total sailing distance of a ship to the straight distance between its departure and destination. The total trajectory sinuosity is calculated by the following formula:

(1) $$\mathrm{sinuosity}=\frac{\sum_{i=1}^{n-1}\mathrm{distance}\left(p\left(i\right),p\left(i+1\right)\right)}{\mathrm{distance}\left(p1,pn\right)}$$

(2) Stop features are the trajectory features extracted from the sub-trajectories in the Stop Mode. The stop features extracted in this paper include two parameters, that is, stop duration and stop range. Stop duration refers to the period of time from the start to the end of a sub-trajectory in the Stop Mode. Stop range is represented by the area of circle of uncertainty for all trajectory points available in a sub-trajectory in the Stop Mode. It is assumed that the stop sub-trajectory is extracted as StopT = {*p*1, *p*2, ……, *pn*}, the central point of stop C($\bar{x}$, $\bar{y}$)is as follows:

Let the matrix stop error radius $R_{e}$ be the maximum distance between all trajectory points in the trajectory segment and the stop central point, there is:

(2) $$R_{e}=\max{\left\{\mathrm{distance}\left(p_{i},\;C\right)\right\}}_{\;\;i=0}^{N}$$

Then the stop range *Sa* is:

(3) $$S_{a}=\pi \cdot R_{e}^{2}$$

(3) Line features are the trajectory features extracted from the sub-trajectories in the Line Mode. The line features extracted in this paper involve three parameters, that is, speed, acceleration, and heading. The “global features” for these parameters are calculated with seven statistical quantities including mean, median, standard deviation, average of three largest numbers, coefficient of variation, skewness, and kurtosis. Among them, skewness is a coefficient for measuring the deviation of data distribution from the symmetrical center and is normally represented by the ratio of three-order center distance to the third power of standard deviation. Kurtosis is a coefficient reflecting the aggregation level of data at the center and normally denoted by the ratio of four-order center distance to the fourth power of standard deviation. Skewness $S_{k}$ and kurtosis $K_{u}$ are calculated as follows:

(4) $$S_{k}=\frac{\sum {\left(x_{i}-\mu \right)}^{3}}{N\cdot \sigma^{3}}$$

(5) $$K_{u}=\frac{\sum {\left(x_{i}-\mu \right)}^{4}}{N\cdot \sigma^{4}}$$

(4) Turn features are the trajectory features extracted from the sub-trajectories in the Turn Mode. In this paper, the extracted turn features involve three parameters in total, that is, angular speed, turn speed, and turn angle. Angular speed is the ratio of the direction difference between two trajectory points and the time. Turn speed is the ratio of the distance between two trajectory points and the time. Turn angle is the difference between the destination direction and the departure direction of all sub-trajectories in the Turn Mode.

### 5.3. Ship Trajectory Classification Based on Motion Features

**Figure 9:**
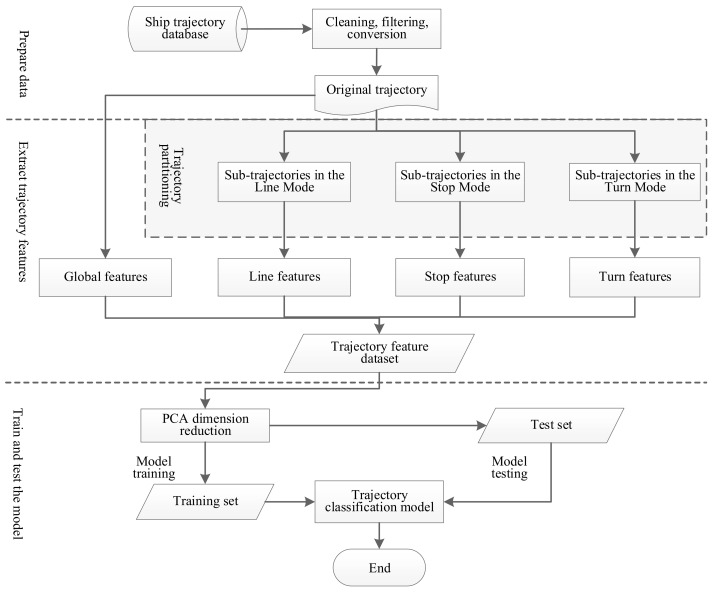
Overall framework of ship trajectory classification model.

Based on the ship trajectory partitioning and trajectory feature extraction, the classification algorithm in the field of machine learning is employed to obtain a ship trajectory classification model through training. The feature extraction method proposed in this paper is taken to construct the overall framework of a ship trajectory classification model as shown in Figure 9.

**Figure 10:**
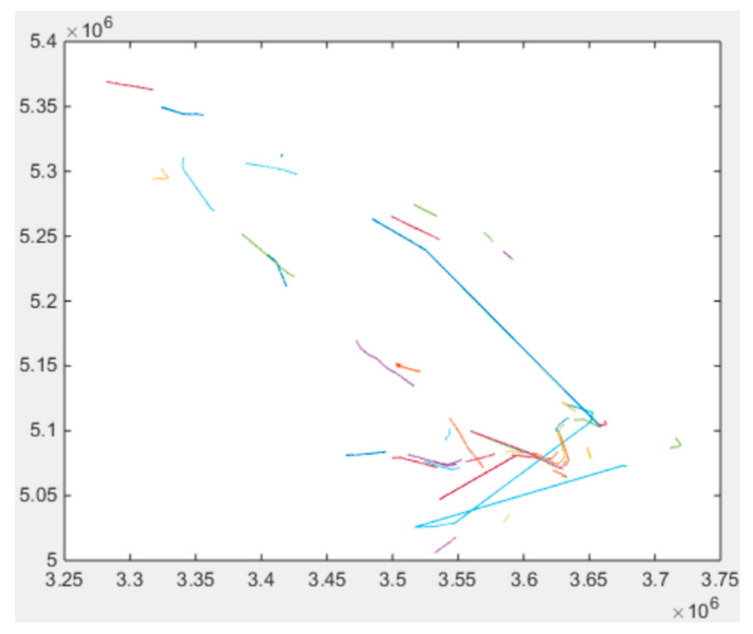
Spatial distribution of ship historical trajectories (50 items).

In this paper, the real AIS data of ships were taken from the ship historical temporal and spatial trajectory library to extract 1000 effective trajectories of fishing boats and cargo ships (500 each) through data cleaning, filtering, conversion, and preliminary processing, for training and testing of the ship classification model [13]. The spatial distribution of the selected ship historical trajectories (50 items) is shown in Figure 10.

Based on the proposed ship trajectory feature extraction method, 158 trajectory features were extracted from the historical trajectories of fishing boats and cargo ships, including global features, stop features, line features, and turn features of each trajectory. Subsequently, the data sets of trajectory features for fishing boats and cargo ships were created together with the statistics and analysis of features.

**Figure 11:**
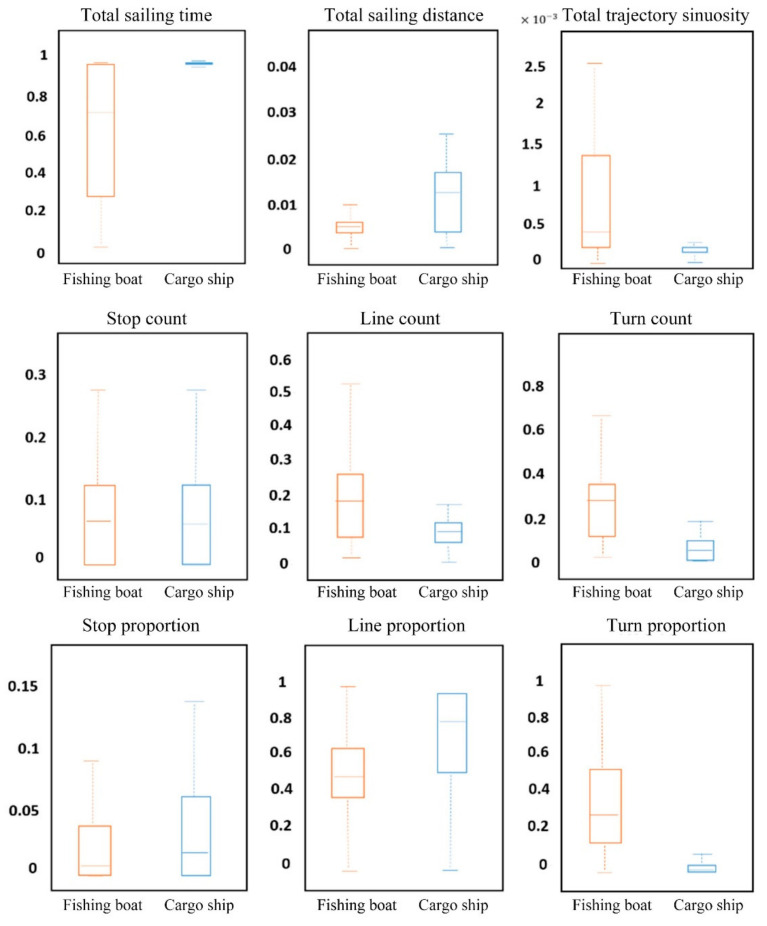
Comparison of global features of fishing boat (blue) and cargo ship (red) trajectories.

As shown in Figure 11, the cargo ship has a longer and more centrally distributed sailing time and a significantly higher proportion of Line Mode than the fishing boat. However, the fishing boat has a larger turn count and line count (i.e., the number of sub-trajectories in the Turn Mode and Line Mode) and a higher proportion of Turn Mode than the cargo ship. The motion pattern of trajectory is not considered for the global features of trajectory in the calculation of total sailing distance and total sailing sinuosity. This may be the reason for some remaining outliers or outlier sub-trajectories. After normalization, these two features are dramatically reduced and not effectively reflected in the box plot.

### 5.4. Ship Classification Model Training and Testing

To verify the effectiveness of the proposed ship trajectory feature extraction method, some popular single classifier learning algorithms in the machine learning field were selected in this paper for training and testing the ship classification model, including Decision Tree (DT), Naïve Bayers (NB), Logistic Regression (LR), Artificial Neural Networks (ANN), and Support Vector Machine (SVM) [21]. The test was implemented with the Python programming language and the scientific computing environment Anaconda. The classification algorithm used the standard model given in the machine learning kit scikit-learn. The default parameters were employed. The classification model was constructed and tested in the following procedure:

(1) Feature selection and dimension reduction:

The principal component analysis (PCA) method [22] was adopted to select the features from the extracted trajectory features for dimension reduction. The threshold for retaining principal components was 95%. After dimension reduction, the number of extracted features is as shown in Table 1.

(2) Classification of training set and test set:

The feature data set was partitioned with hold-out. Before each test, the feature data set was randomly divided into two mutually exclusive sets, that is, 75% training set and 25% test set.

(3) Model training and evaluation:

Two indicators were selected to evaluate the results of model prediction, that is, accuracy and area under curve (AUC) [22]. For this binary problem, the model predicted results and actual results of the test set form a confusion matrix presented in Table 2.

Accuracy indicates the proportion of all predicted results as follows:

(6) $$\mathrm{Accuracy}=\frac{TP+TN}{TP+FN+FP+FN}$$

The receiver operating characteristic curve (ROC) can be used to reflect the performance of model classification. The true positive rate (*TPR*) and false positive rate (*FPR*) are as follows:

(7) $$TPR=\frac{TP}{TP+FN}$$

(8) $$FPR=\frac{FP}{TN+FP}$$

**Figure 12:**
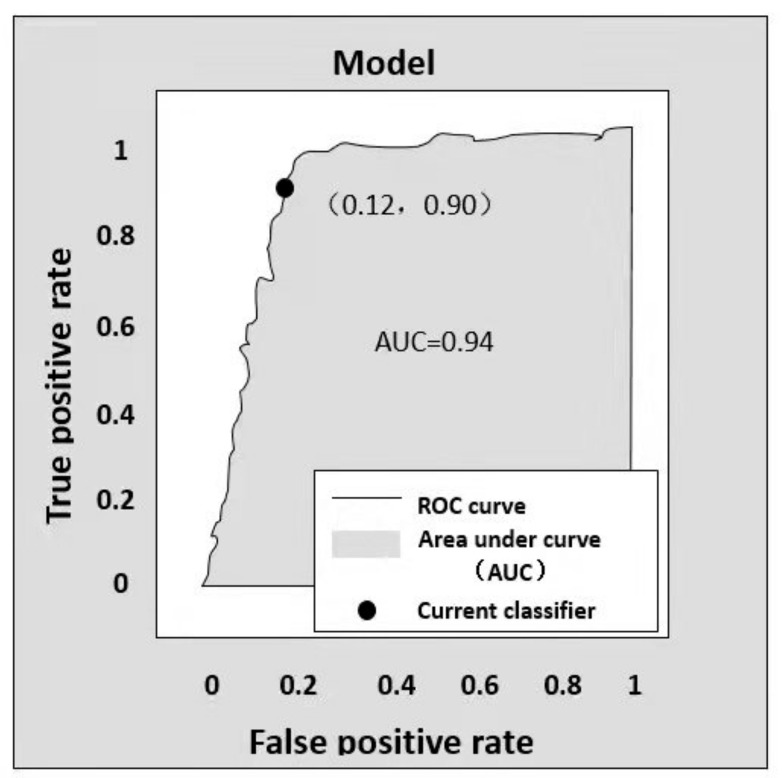
Diagram of ROC curve and AUC.

Based on the predicted results of classifiers, samples were sequenced and correspondingly predicted as the positive class. Their *FPR* and *TPR* were calculated, respectively, and then used to obtain the ROC curve with *FPR* as the horizontal axis and *TPR* as the longitudinal axis, as shown in Figure 12. Normally, the closer the ROC curve gets to the upper left corner, the better performance of a classifier. AUC is the area encircled under the ROC curve. It is a quantitative description in place of the ROC curve. AUC ranges between 0 and 1. The larger the AUC, the better performance of a classifier.

**Figure 13:**
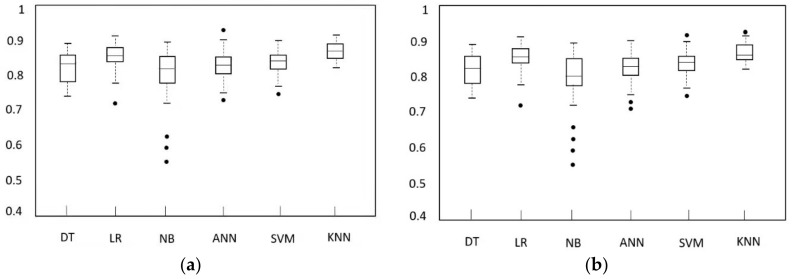
Comparison of the distribution of evaluation indicators of each classifier. (**a**) Comparison of classification accuracy of each classifier; (**b**) Comparison of AUC scores of each classifier.

The average accuracy and AUC values of the classification model are indicated in Table 3. The specific distribution is given in Figure 13. Figure 13a shows the accuracy distribution of the classification model from 100 independent tests. Figure 13b provides the AUC distribution of the model.

The analysis reveals that the prediction performance of the trained model was dramatically improved when the trajectory was reasonably partitioned and then trajectory features were extracted from sub-trajectories. In the meantime, features were extracted with the proposed ship trajectory partitioning method in terms of motion mode to further enhance the prediction performance of classifiers.

## 6. System Implementation

Presently, the ship trajectory management and analysis system (STDMAS) has implemented such functions as identification of unknown ships and ship outlier detection in the database management module, trajectory analysis model, and trajectory mining module [29]. In this section, the functional modules of STDMAS are illustrated with some images including the main interfaces of system management configuration, database management, trajectory analysis, trajectory mining, and situation analysis.

### 6.1. Database Management Module

**Figure 14:**
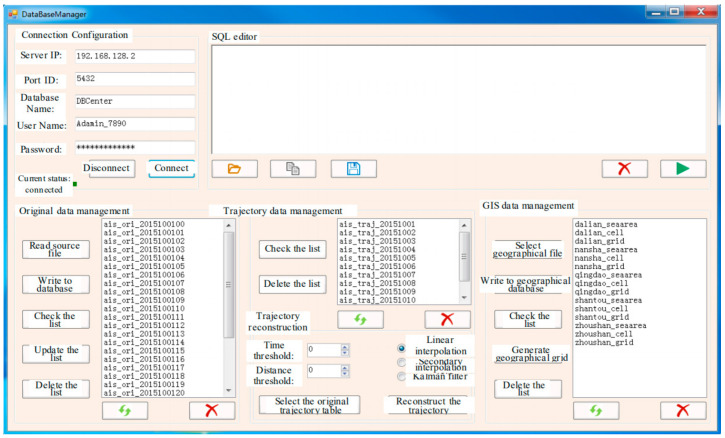
STDMAS database management interface.

With the database management of the system, a user can read the original files of original maritime data, trajectory data, and GIS data, and can also convert, write, check, update, and delete the data from the database, as shown in Figure 14. When reconstructing the trajectory data, the parameters including time threshold and distance threshold can be set to filter the outliers in the original data. Additionally, interpolation or filter algorithms may be chosen to process the data as needed [30].

### 6.2. Trajectory Analysis Module

**Figure 15:**
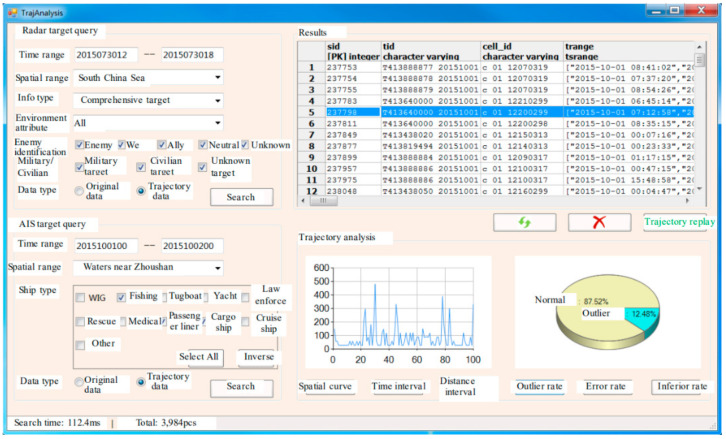
STDMAS trajectory analysis interface.

**Figure 16:**
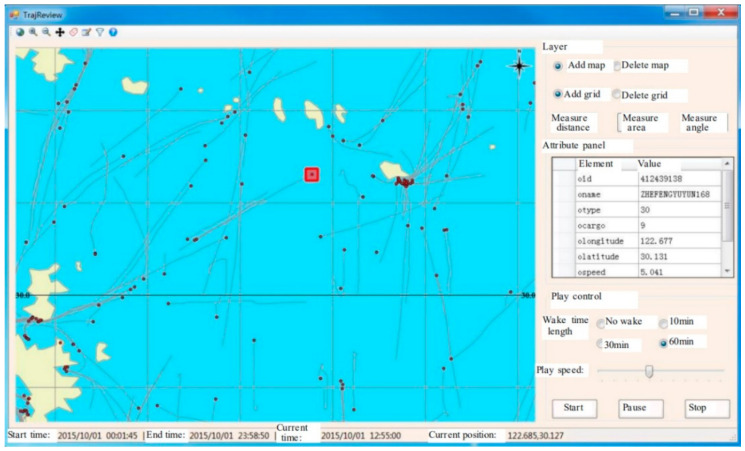
STDMAS historical trajectory review interface.

This module can implement the fast query of radar detection or AIS target original data and trajectory target within the spatial and time ranges and analyze the variation tendency of the spatial curve, sampling time interval, and distance interval of trajectories [6], as shown in Figure 15. It can also calculate the outlier rate, error rate, and inferior rate of the radar detection data, so as to indirectly reflect the target detection performance of radar. Moreover, the trajectory analysis module of STDMAS is integrated with the review and replay feature of historical trajectories to review and analyze the historical situation of military exercises, major missions, or other special maritime activities. The functions of the module are as shown in Figure 16. The interface integrates the common measuring tools for distance, area, and angle, etc., in the GIS software to facilitate the user’s analysis and calculation. A user may select and control the retention length of target wake and the replay speed of historical situation in this interface, so as to implement the detailed review and analysis of historical maritime situations [31].

### 6.3. Trajectory Mining Module

Based on the abovementioned trajectory data mining method, the trajectory mining module of STDMAS can implement the identification of unknown targets and the outlier detection of targets. However, both functions depend on the training and implementation of the trajectory classification model [32].

**Figure 17:**
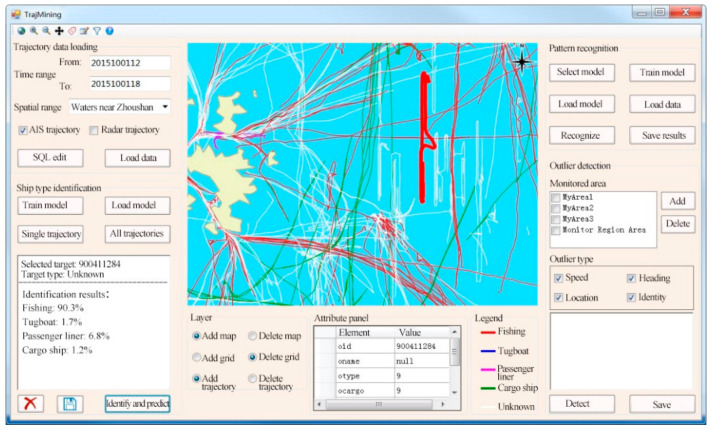
STDMAS unknown ship identification interface.

Figure 17 presents the unknown ship identification function of the STDMAS trajectory mining module. The “trajectory data loading” panel in the upper left of the interface is used to check the ship trajectory within the given time or space ranges. All the searched trajectories are displayed in the GIS display module in the middle of the interface. The “ship type identification” panel in the lower left of the interface can load the trained trajectory classification model and identify the selected unknown ship trajectory. In the figure, an unknown ship trajectory with the target No. 900,411,284 is selected, and its ship identification and prediction results include fishing: 90.3%, tugboat: 1.7%, passenger liner: 6.8%, and cargo ship: 1.2%. Hence, the target is identified as a fishing boat in the system.

**Figure 18:**
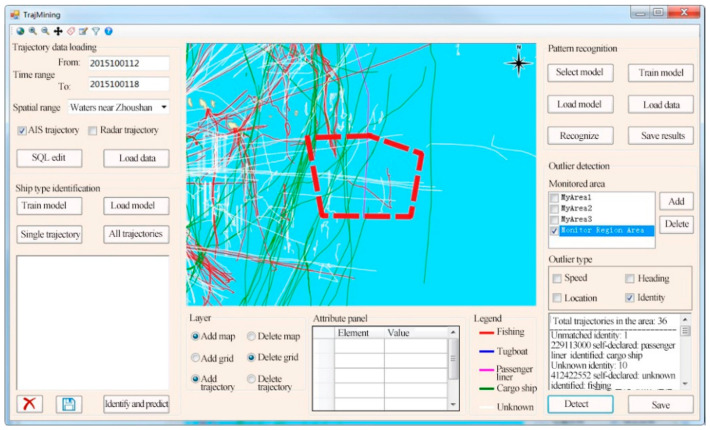
STDMAS ship identity outlier detection interface.

Figure 18 presents the outlier detection function of the STDMAS trajectory mining module. The buttons “Add” and “Delete” in the “outlier detection” panel in the right section of the interface are used to implement the setting and management of the monitored area. The tabs “Monitored Area” and “Outlier Type” are used to select the area to be monitored (for outlier detection) and the type of outlier. The text box in the lower right of the interface displays the system’s target identity outlier detection results in the “Monitor Region Area”.

## 7. Conclusions

To address the data storage, management, analysis and mining, of maritime targets, an object-oriented method was adopted to design the overall structure and functional modules of the ship trajectory management and analysis system (STDMAS). This paper elaborates the design and technical details of the STDMAS functional modules including logical structure, module composition, physical deployment, database management, trajectory analysis, trajectory mining, and situation analysis. A ship identification method based on motion features was put forward. With the proposed method, ship trajectory was first partitioned into sub-trajectories in various behavioral patterns, and effective motion features were then extracted. Machine learning algorithms were utilized for training and testing to identify many types of ships. The functional modules implemented for the system include database management, trajectory analysis, historical situation review, ship identification, and outlier detection based on trajectory classification. STDMAS can satisfy the practical needs for the data management, analysis, and mining of maritime targets because it is easy to apply, maintain, and expand. Efforts will be made to integrate such functions as trajectory cluster analysis and mining and trajectory situation analysis in STDMAS. Moreover, STDMAS will be connected to the real-time maritime target data receiving system, and then tested to further provide the data support and computation service for research and application personnel in the management, analysis, and mining of maritime target data. Additionally, the system can process a limited size of AIS data at present, so that big data algorithm and cloud computing architecture may be employed to improve the efficiency of mass data processing algorithms in future research.

## Figures and Tables

**Table 1:** Selected features using PCA.

Number of original features	158
Number of features after dimension reduction	24

**Table 2:** Confusion matrix.

Actual Class	Predicted Result	
	Positive Class	Negative Class
Positive class	True positive (*TP*)	False Negative (*FN*)
Negative class	False Positive *(FP*)	True Negative (*TN*)

**Table 3:** Comparison of classification performance of classifiers.

Classification Model	Evaluation Indicator	Result
Decision tree (DT)	Accuracy	0.8560
	AUC	0.8558
Naïve Bayers (NB)	Accuracy	0.8443
	AUC	0.8435
Logistic Regression (LR)	Accuracy	0.8837
	AUC	0.8833
Artificial Neural Networks (ANN)	Accuracy	0.8260
	AUC	0.8159
Support Vector Machine (SVM)	Accuracy	0.8456
	AUC	0.8451
K-nearest Neighbor (KNN)	Accuracy	0.8379
	AUC	0.8377

## Data Availability

Not applicable.
